# Social Factors Associated With Nutrition Risk in Community-Dwelling Older Adults in High-Income Countries: Protocol for a Scoping Review

**DOI:** 10.2196/56714

**Published:** 2024-06-25

**Authors:** Christine Marie Mills, Liza Boyar, Jessica A O’Flaherty, Heather H Keller

**Affiliations:** 1 Department of Kinesiology and Health Sciences University of Waterloo Waterloo, ON Canada; 2 School of Public Health Sciences University of Waterloo Waterloo, ON Canada; 3 Mount Saint Vincent University Halifax, NS Canada; 4 Schlegel-UW Research Institute for Aging Waterloo, ON Canada

**Keywords:** community, malnutrition risk, nutrition risk, older adults, social factors, geriatric, geriatrics, malnutrition, community-dwelling, older adult, elderly, HIC, high-income countries, diet, dietary intake, nutritional status, Canada, nutritional risk, social, intervention, public health, community-based intervention, health promotion

## Abstract

**Background:**

In high-income countries (HICs), between 65% and 70% of community-dwelling adults aged 65 and older are at high nutrition risk. Nutrition risk is the risk of poor dietary intake and nutritional status. Consequences of high nutrition risk include frailty, hospitalization, death, and reduced quality of life. Social factors (such as social support and commensality) are known to influence eating behavior in later life; however, to the authors’ knowledge, no reviews have been conducted examining how these social factors are associated with nutrition risk specifically.

**Objective:**

The objective of this scoping review is to understand the extent and type of evidence concerning the relationship between social factors and nutrition risk among community-dwelling older adults in HICs and to identify social interventions that address nutrition risk in community-dwelling older adults in HICs.

**Methods:**

This review will follow the scoping review methodology as outlined by the JBI Manual for Evidence Synthesis and the PRISMA-ScR (Preferred Reporting Items for Systematic Reviews and Meta-Analyses extension for Scoping Reviews) guidelines. The search will include MEDLINE (Ovid), CINAHL, PsycINFO, and Web of Science. There will be no date limits placed on the search. However, only resources available in English will be included. EndNote (Clarivate Analytics) and Covidence (Veritas Health Innovation Ltd) will be used for reference management and removal of duplicate studies. Articles will be screened, and data will be extracted by at least 2 independent reviewers using Covidence. Data to be extracted will include study characteristics (country, methods, aims, design, and dates), participant characteristics (population description, inclusion and exclusion criteria, recruitment method, total number of participants, and demographics), how nutrition risk was measured (including the tool used to measure nutrition risk), social factors or interventions examined (including how these were measured or determined), the relationship between nutrition risk and the social factors examined, and the details of social interventions designed to address nutrition risk.

**Results:**

The scoping review was started in October 2023 and will be finalized by August 2024. The findings will describe the social factors commonly examined in the nutrition risk literature, the relationship between these social factors and nutrition risk, the social factors that have an impact on nutrition risk, and social interventions designed to address nutrition risk. The results of the extracted data will be presented in the form of a narrative summary with accompanying tables.

**Conclusions:**

Given the high prevalence of nutrition risk in community-dwelling older adults in HICs and the negative consequences of nutrition risk, it is essential to understand the social factors associated with nutrition risk. The results of the review are anticipated to aid in identifying individuals who should be screened proactively for nutrition risk and inform programs, policies, and interventions designed to reduce the prevalence of nutrition risk.

**International Registered Report Identifier (IRRID):**

DERR1-10.2196/56714

## Introduction

In high-income countries (HICs), between 65% and 70% of community-dwelling adults aged 65 years and older are at high nutrition risk [1]. In Canada, one-third of community-dwelling adults aged 60 and older are at increased nutrition risk [2,3]. While there is no universally agreed-upon definition of nutrition risk, 1 common definition is the risk of poor dietary intake and nutritional status [3,4]. Nutrition risk occurs when there are factors present that negatively affect food intake [5], and can lead to malnutrition if its causes are not addressed [3].

**Figure 1:**
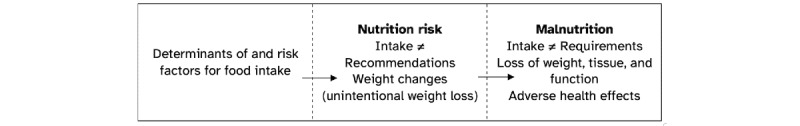
The conceptual relationship between nutrition risk and malnutrition, adapted from Keller [8] with permission from Heather H Keller.

Many measures or instruments, commonly referred to as tools, are available to measure and screen for nutrition risk and malnutrition risk in community-dwelling older adults, such as Seniors in the Community Risk Evaluation for Eating and Nutrition and the Mini Nutritional Assessment [6]. Nutrition risk and malnutrition risk lie on a continuum between nutritional health and malnutrition [3]. Malnutrition is a clinical condition that occurs when there is unintentional weight loss, low body mass index, or reduced muscle mass, along with reduced food intake, reduced assimilation of food and nutrients, chronic disease, or inflammation related to acute disease or injury [7]. Malnutrition risk exists in clinical settings when individuals have 1 or more of these indicators [8]. In contrast, nutrition risk occurs earlier in the process. Nutrition risk “represents the determinants and risk factors that place an individual at risk for poor food intake and if not interrupted, can lead to malnutrition” [8]. However, in the literature, the terms nutrition risk and malnutrition risk are often used interchangeably, with nutrition risk sometimes referred to as early-stage malnutrition risk [9]. The following figure, adapted from Keller [8], illustrates the relationship between food intake, nutrition risk, and malnutrition (Figure 1).

Individuals who are at nutrition risk are more likely to be hospitalized and have an increased risk of mortality, even when health status, health behaviors, and socioeconomic factors are considered [10,11]. Nutrition risk is also associated with early institutionalization [12], frailty [13], and poor quality of life [14,15].

Many social factors are associated with nutrition risk in older adults in the literature [16,17]. These include, but are not limited to, social network type (restricted, diverse, family-focused, friend-focused, etc), living arrangement (alone or with others), social support (emotional, informational, tangible, and affectionate support), social engagement (engagement in social activities with others), and social participation (participation in community activities) [3,17-19]. It is well-established that eating with others improves dietary intake and reduces nutrition risk [16], whereas eating alone is associated with high nutrition risk [16,20]. In many studies, social isolation is a significant factor leading to increased nutrition risk [3,11]. Social relationships may decrease nutrition risk by encouraging compliance with dietary norms [16], while eating with others may provide “social cues for when and what to eat” [16]. An individual’s social support system may also encourage healthy behaviors, such as consuming adequate amounts of nutrient-rich foods [21]. Studies have found that social support helps reduce nutrition risk [17], whereas low levels of social support are associated with increased nutrition risk [22]. Individuals with higher levels of social support may have greater assistance with food-related activities, such as meal preparation and grocery shopping [16]. Social interventions, such as congregate meal programs, have also been shown to improve nutrition risk scores in older adults. Despite the research showing that social factors are associated with nutrition risk, reviews in this area have not specifically focused on nutrition risk, but rather on eating behavior [16], dietary intake [23], food choice [24], or nutrition screening [25]. Although related to these concepts, nutrition risk is different, describing the presence of risk factors and determinants of food intake such as poor appetite, food insecurity, and low dietary intake [14,26].

To advance research on how social factors are associated with nutrition risk, it is essential to synthesize the available evidence. A scoping review was chosen as it is an appropriate method to understand and summarize the extent and type of evidence available [27]. It is important to understand the social factors associated with nutrition risk so that individuals who should be screened proactively for nutrition risk can be identified, and programs and policies designed to reduce the prevalence of nutrition risk can be appropriately developed and implemented. Understanding what types of social interventions have been shown to reduce the prevalence of nutrition risk can also help inform such policy and program development.

This review aims to examine the social factors associated with nutrition risk in community-dwelling older adults in HICs and to identify social interventions designed to address nutrition risk in community-dwelling older adults in HICs. A preliminary search was conducted in October 2023 to identify any existing systemic or scoping reviews on this topic. Searching PROSPERO, MEDLINE (Ovid), the Cochrane Database of Systematic Reviews, and JBI Evidence Synthesis identified no current or in-progress scoping reviews or systematic reviews on the topic. There have been reviews examining adjacent topics, for example, the social influences on eating behavior in later life [16], factors influencing food choice in older adults [24], and determinants of dietary intake in older people [23] although none of these have specifically examined the relationship between nutrition risk and social factors or examined social interventions designed to address nutrition risk.

The results of this scoping review will provide researchers and clinicians with knowledge of the social factors associated with nutrition risk in community-dwelling older adults in HICs. Identifying the social factors associated with nutrition risk can help inform future research into nutrition risk and guide program and policy development. Promising social interventions that can improve nutrition risk may also be identified. Additionally, the results of this review may help identify groups of individuals who should be screened for nutrition risk.

## Methods

### Scoping Review Methodology

The proposed scoping review will be conducted in accordance with the JBI methodology for scoping reviews [28] and in line with the PRISMA-ScR (Preferred Reporting Items for Systematic Reviews and Meta-analyses extension for Scoping Reviews) [29]***.***

### Research Questions

The research questions for this scoping review are as follows: (1) What social factors (including, but not limited to, social networks, social engagement, social support, and social participation) are associated with nutrition risk or malnutrition risk among community-dwelling adults aged 60 years and older living in HICs? (2) What social interventions improve nutrition risk or malnutrition risk in community-dwelling adults aged 60 years and older living in HICs?

### Search Strategy

The search strategy was developed with the assistance of a university research librarian and will be conducted electronically. The search will aim to locate both published and unpublished studies and reviews. An initial limited search of MEDLINE and CINAHL was undertaken to identify articles on the topic. The initial search terms included “malnutrition,” “nutrition risk,” “older adults,” “social factors,” “social support,” “social engagement,” “social participation,” “social influence,” and “social networks.” The text words contained in the titles and abstracts of relevant articles and the index terms used to describe the articles were used to develop a full search strategy for MEDLINE, CINAHL, PsycINFO, and Web of Science. A sample search strategy for MEDLINE is available in Table 1.

**Table 1:** Sample search strategy for a scoping review examining social factors and nutrition risk in community-dwelling older adults in high-income countries; results of a search of MEDLINE on October 10, 2023.

Search	Query	Records retrieved, n
#1	exp Malnutrition/ or exp Nutritional Status/ or nutrition risk.mp.	167,909
#2	undernutrition.mp.	8345
#3	poor dietary intake.mp.	227
#4	poor nutrition.mp. or exp Diet/	305,502
#5	1 or 2 or 3 or 4:	453,221
#6	exp Aged/ or exp Aging/ or older adults.mp.	3,506,670
#7	seniors.mp.	8211
#8	elderly.mp.	277,535
#9	6 or 7 or 8:	3,574,189
#10	social factors.mp. or exp Social Factors/	11,115
#11	social support.mp. or exp Social Support/	98,186
#12	social engagement.mp. or exp Social Participation/	4565
#13	social influence.mp. or exp Social Behavior/	276,421
#14	social networks.mp. or exp Social Networking/	14,147
#15	10 or 11 or 12 or 13 or 14:	383,726
#16	5 and 9 and 15:	874

The search strategy, including all identified keywords and index terms, will be adapted for each included database or information source. The reference lists of articles included in the review will be screened for additional studies. Only studies in the English language will be included, as there are limits to language proficiency within the research team. No date restrictions will be applied when conducting searches. As recommended by a research librarian, sources of unpublished studies or gray literature will be searched, including preprint servers such as medRxiv, ProQuest dissertations and theses, Open Grey, and Google Scholar. Both social factors associated with nutrition risk and social interventions designed to address nutrition risk will be searched for in the gray literature.

### Study Selection

#### Overview

Inclusion and exclusion criteria have been developed according to the domains of participants, concept, context, and types of evidence sources, as recommended in the JBI Manual for Evidence Synthesis [28]. The inclusion and exclusion criteria (Textbox 1) are described in detail.

**Inclusion and exclusion criteria for screening sources of evidence for a scoping review examining social factors and nutrition risk in community-dwelling older adults in high-income countries.**

**Inclusion criteria**

- Older adults (60 years of age and older)
- Community-dwelling
- High-income countries
- Social factors including social support, social engagement, social participation, social isolation, social inclusion, commensality (eating with others), and loneliness
- Social interventions such as congregate meals programs, group nutrition education programs, etc
- Nutrition risk, malnutrition risk, and risk of malnutrition

**Exclusion criteria**

- Age groups other than older adults (unless information for older adults available separately)
- Long-term care, care homes, hospital, institutionalized, and assisted living
- Low- or middle-income countries
- No social factors in relation to nutrition risk
- Not a social intervention
- Other nutritional factors such as risk of overnutrition, disease-related malnutrition, diet quality, diet patterns, nutrition risk in specific diseases (ie, diabetes, heart disease, kidney disease, dementia, cancer, and COVID-19)

#### Inclusion Criteria

##### Participants

This review will consider studies that include community-dwelling adults aged 60 years and older in HICs. The minimum participant age of 60 years is consistent with the United Nations’ definition of older persons [30,31]. Studies that include other age groups but present the results of adults aged 60 years and older as a subgroup will be included, whereas studies that do not separate this age group will not be included, as the causes of nutrition risk may differ in younger age groups. Similarly, studies that examine disease-related nutrition risk and malnutrition risk will be excluded from this review, as specific diseases affect nutrition risk and malnutrition risk in different ways.

This review will be restricted to HICs, given that there is a paucity of literature examining nutrition status specifically in older adults living in low- and middle-income countries (LMICs) [32], and that the causes of nutrition risk are likely different in LMICs compared with HICs [33]. In LMICs, food insecurity and rapid urbanization are the major contributors to malnutrition in older adults [32], and access to sufficient health care and welfare may not be adequate [34]. Moreover, adherence to the use of validated malnutrition risk screening and assessment tools is low in LMICs, rendering the prevalence of nutrition risk among older adults in these settings largely unquantified [32].

##### Concept

This review will consider studies that explore nutrition risk and malnutrition risk, in the community. While there is no universally agreed-upon definition of nutrition risk or malnutrition risk, 1 common definition is the risk of poor dietary intake and nutritional status that places an individual at risk of developing malnutrition if action is not taken to improve dietary intake [3,4]. Nutrition risk occurs when there are factors present that negatively affect food intake or nutrient metabolism [5].

##### Context

This review will consider studies that examine social factors and social interventions. Social factors include, but are not limited to, social support, social networks, social engagement, and social participation. Social networks are “the web of social relationships that surround an individual and the characteristics of those ties” [35]. Social participation refers to participation in activities with friends and family or the community [36], whereas social engagement emphasizes meaningful engagement and desire for social change [37]. Social support includes “the functions that individuals in a person’s social network perform” [36]. The term “social factors” can be said to encompass these concepts.

##### Types of Evidence Sources

This scoping review will consider both experimental and quasi-experimental study designs, including randomized controlled trials that examine how social factors or social interventions affect nutrition risk, nonrandomized controlled trials, before and after studies, and interrupted time-series studies. In addition, analytical observational studies, including prospective and retrospective cohort studies, case-control studies, and analytical cross-sectional studies, will be considered for inclusion. This review will also consider descriptive observational study designs including case series, individual case reports, and descriptive cross-sectional studies for inclusion.

Qualitative studies will also be considered, including, but not limited to, designs such as phenomenology, grounded theory, ethnography, qualitative description, action research, and feminist research. In addition, systematic reviews, rapid reviews, and narrative reviews will be considered for inclusion in the proposed scoping review. Papers that are conference abstracts or letters to the editor will not be considered for inclusion in this scoping review.

Gray literature consisting of dissertations, theses, reports, or unpublished papers that examine how social factors affect nutrition risk or that describe social interventions designed to address nutrition risk will also be considered for inclusion.

### Study or Source of Evidence Selection

Following the search, all identified citations will be collated and uploaded into EndNote 21 and duplicates will be removed. Citations will then be uploaded to Covidence. Following a pilot test, titles and abstracts will then be screened by 2 or more independent reviewers for assessment against the inclusion criteria for the review. Potentially relevant sources will be retrieved in full, and their full texts will be imported into Covidence. The full text of selected citations will be assessed in detail against the inclusion criteria by 2 independent reviewers. Reasons for the exclusion of sources of evidence in full text that do not meet the inclusion criteria will be recorded and reported in the scoping review. Any disagreements that arise between the reviewers at each stage of the selection process will be resolved through discussion, or with additional reviewers. The results of the search and the study inclusion process will completely be reported in the final scoping review and presented in the PRISMA-ScR flow diagram [29].

### Data Extraction

Data will be extracted from sources of evidence included in the scoping review by 2 independent reviewers, using an adaptation of the Covidence data extraction tool as shown in Textbox 2. The extraction tool is based on the JBI guidelines for data extraction and the default Covidence extraction tool. The data extracted will include specific details about the participants, concept, context, study methods, and key findings relevant to the review questions (social factors associated with nutrition risk). Data will be extracted verbatim from the included sources.

The data extraction tool will be further modified and revised, if necessary, during the process of extracting data from each included evidence source. Modifications will be detailed in the scoping review. Any disagreements that arise between the reviewers will be resolved through discussion, or with an additional reviewer. If appropriate, authors of papers will be contacted to request missing or additional data, where required.

**Data extraction instrument for a scoping review examining social factors associated with nutrition risk in community-dwelling older adults in high-income countries.**

**General information**

- Title
- Authors
- Journal
- Year
- Country

**Study characteristics**

- Study aims
- Study design
- Study methods
- Dates (start date, end date, and length of follow-up period for longitudinal studies)
- Study funding sources
- Possible conflicts of interest for study authors

**Participants**

- Population description
- Inclusion criteria for study
- Exclusion criteria for study
- Recruitment methods
- Total number of participants
- Baseline population characteristics

**Main findings**

- Nutrition risk, malnutrition risk measure, or tool
  SCREEN-3 (Seniors in the Community Risk Evaluation for Eating and Nutrition-3)
  SCREEN-8 (Seniors in the Community Risk Evaluation for Eating and Nutrition-8, formerly SCREEN II AB)
  SCREEN-14 (Seniors in the Community Risk Evaluation for Eating and Nutrition-14, formerly SCREEN II)
  SCREEN/SCREEN I (Seniors in the Community Risk Evaluation for Eating and Nutrition/Seniors in the Community Risk Evaluation for Eating and Nutrition I)
  MNA (Mini Nutritional Assessment)
  MNA-SF (Mini Nutritional Assessment-Short Form)
  SGA (Subjective Global Assessment)
  DETERMINE (Determine your health checklist)
  SNAQ (Short Nutritional Assessment Questionnaire)
  MUST (Malnutrition Universal Screening Tool)
  Other (describe)
- Social factors included
  Social support
  Social influence
  Social participation
  Commensality (eating with others)
  Living situation (alone or with others)
  Social networks or social network types
  Social capital
  Social standing
  Social isolation
  Loneliness
  Other (describe)
- Measures of social factors (social factor and how it was measured)
- Found a relationship between social factors and malnutrition and nutrition risk (yes, no, or other)
- Description of the relationship between social factors and malnutrition and nutrition risk

**Additional information for intervention studies**

- Description of intervention
- Time points (number and time between time points)
- Outcomes at different time points

**Additional information for trials**

- Description of control group
- Comparison of intervention group to control group

### Data Analysis and Presentation

Social factors associated with nutrition risk or malnutrition risk will be reported in the scoping review, as will any social interventions that affect nutrition risk or malnutrition risk. Data will be analyzed to identify the common social factors that have been examined in conjunction with nutrition risk and to identify which social factors and interventions have been found to affect nutrition risk. Similarities and differences between findings will be noted; for example, differences in the tools used to measure nutrition risk, differences in how the various social factors were measured, and whether a social factor was consistently associated with nutrition risk across all (or the majority of) studies. Data from studies included in this review will be presented as a narrative summary with the use of tables. The tables will be used to highlight central concepts described in the narrative summary.

## Results

The scoping review was started in October 2023 and will be finalized by August 2024. As of May 28, 2024, a total of 5064 studies have been identified through database searches and imported for title and abstract screening, and 293 duplicates have been removed (266 duplicates identified by Covidence and 27 duplicates identified manually). Title and abstract screening has been completed, resulting in 235 full-text studies ready to be assessed for eligibility. The results of this scoping review will provide a comprehensive understanding of the social factors associated with nutrition risk in community-dwelling older adults in HICs.

## Discussion

### Expected Outcomes

This scoping review will identify which social factors have been examined concerning nutrition risk in the literature and which social factors have been shown to impact nutrition risk. It will also identify any social interventions that have successfully reduced the prevalence of nutrition risk. The results of the review are anticipated to aid in identifying individuals who should be screened proactively for nutrition risk by identifying the social factors associated with increased risk. If the literature shows that certain social factors are consistently associated with increased nutrition risk, then screening programs can target individuals who reflect those social factors, for example, individuals with low levels of social support [17,22], low levels of social participation [3,17], or those who are socially isolated [3,11,22,38].

The results of this review will also inform programs and policies designed to reduce the prevalence of nutrition risk and help guide the development of interventions aimed at reducing nutrition risk through the identification of interventions that have affected nutrition risk and social factors that impact nutrition risk.

Gaps in the literature exploring social factors associated with nutrition risk will also be identified by noting which social factors have not been well-studied in the literature. This will help inform future research into nutrition risk and social factors.

### Strengths and Limitations

A strength of this scoping review is the use of the JBI methodology for scoping reviews [28], and the PRISMA-ScR [29]. All screening and data extraction will be completed by at least 2 reviewers, out of whom at least 1 is a registered dietitian with experience in working with older adults in the community. Any disagreements between the reviewers will be resolved through discussion or by a third reviewer.

Due to many different tools that can be used to measure nutrition risk in community-dwelling older adults and due to the many ways that social factors can be measured, it is anticipated that there will be significant heterogeneity between studies. This information will be captured in the data extraction tool and will be discussed in the scoping review.

### Conclusions

With the high prevalence of nutrition risk in community-dwelling older adults in HICs and the negative consequences of nutrition risk, it is essential to understand the social factors associated with nutrition risk. The Canadian Malnutrition Task Force has identified that research into the root causes of nutrition risk in community-dwelling older adults is a priority [39], and this scoping review will help to identify the potential social factors that may be the root causes of nutrition risk among this demographic.

## Abbreviations

HIC: high-income country
LMIC: low- and middle-income country
PRISMA-ScR: Preferred Reporting Items for Systematic Reviews and Meta-Analyses extension for Scoping Reviews
